# Seven day pre-analytical stability of serum and plasma neurofilament light chain

**DOI:** 10.1038/s41598-021-90639-z

**Published:** 2021-05-26

**Authors:** Patrick Altmann, Markus Ponleitner, Paulus Stefan Rommer, Helmuth Haslacher, Patrick Mucher, Fritz Leutmezer, Axel Petzold, Christoph Wotawa, Rupert Lanzenberger, Thomas Berger, Henrik Zetterberg, Gabriel Bsteh

**Affiliations:** 1 Department of Neurology, Medical University of Vienna, Waehringer Guertel 18-20, 1090 Vienna, Austria; 2 Department of Laboratory Medicine, Medical University of Vienna, Vienna, Austria; 3 Department of Neuroinflammation, UCL Queen Square Institute of Neurology, London, UK; 4 Department of Psychiatry and Psychotherapy, Medical University of Vienna, Vienna, Austria; 5 Department of Neurodegenerative Disease, UCL Queen Square Institute of Neurology, London, UK; 6 UK Dementia Research Institute at UCL, London, UK; 7 Department of Psychiatry and Neurochemistry, Institute of Neuroscience and Physiology, The Sahlgrenska Academy at the University of Gothenburg, Mölndal, Sweden; 8 Clinical Neurochemistry Laboratory, Sahlgrenska University Hospital, Mölndal, Sweden

**Keywords:** Neurology, Neuroimmunology

## Abstract

Neurofilament light chain (NfL) has emerged as a biomarker of neuroaxonal damage in several neurologic conditions. With increasing availability of fourth-generation immunoassays detecting NfL in blood, aspects of pre-analytical stability of this biomarker remain unanswered. This study investigated NfL concentrations in serum and plasma samples of 32 patients with neurological diagnoses using state of the art Simoa technology. We tested the effect of delayed freezing of up to 7 days and statistically determined stability and validity of measured concentrations. We found concentrations of NfL in serum and plasma to remain stable at room temperature when processing of samples is delayed up to 7 days (serum: mean absolute difference 0.9 pg/mL, intraindividual variation 1.2%; plasma: mean absolute difference 0.5 pg/mL, intraindividual variation 1.3%). Consistency of these results was nearly perfect for serum and excellent for plasma (intraclass correlation coefficients 0.99 and 0.94, respectively). In conclusion, the soluble serum and plasma NfL concentration remains stable when unprocessed blood samples are stored up to 7 days at room temperature. This information is essential for ensuring reliable study protocols, for example, when shipment of fresh samples is needed.

## Introduction

Neurofilaments are part of the axon’s cytoskeleton and are released into the blood and cerebrospinal fluid following axonal damage^1,2^. Over the past decade, neurofilament light chain (NfL) has emerged as a multifaceted biomarker in blood allowing for the detection of damage to the central and peripheral nervous system^3–6^. With its ability to predict treatment response and neurological outcome in some cases, NfL has since been coined *the c-reactive protein of neurology*^7^. State of the art single molecule array (Simoa) technology allows for reliable detection of this low-concentrated biomarker in the range of a few picograms per milliliter^8,9^.

With an ever-increasing amount of NfL research and study protocols employing the inclusion of NfL from blood, there is an important pre-analytical consideration for sample storage and handling. Currently, there is some evidence from a small cohort suggesting that *serum* NfL are stable if unprocessed blood is stored up to 7 days at room temperature^10^. Furthermore, a more recent study shows stability for NfL concentrations in serum if samples are left up to 24 h at room temperature^11^. However, there is no investigation with the primary hypothesis of confirming stability of NfL up to 7 days in unprocessed samples of *both* serum and plasma.

This study explores the pre-analytical stability of NfL in serum and plasma with conditions in mind, for which standard processing might not be possible. These circumstances may apply to, for instance, an out of hospital collection requiring shipment to other facilities or sample collection outside of regular working hours. Therefore, the core investigation involved measurement of NfL concentration in serum and plasma from 32 volunteers whose sample processing (i.e., centrifuging and freezing) was delayed up to 7 days. We also report on the analytical performance characteristics of this assay with respect to precision and repeatability.

## Methods

### Ethics statement

The ethics committee at the Medical University of Vienna (protocol number EK2100/2019, amended in July 2020) approved this study. Inclusion of participants into the biobanking protocol followed written informed consent. This study complies with the World Medical Association Declaration of Helsinki regarding ethical conduct of research involving human subjects.

### Study population

For this study, we used serum and plasma samples of participants from our outpatient clinic at the Department of Neurology at the Medical University of Vienna, Austria. To avoid interference with our comparing measurements in serum and plasma, only patients not taking any medication affecting thrombocyte function of blood coagulation were included. Based on a previous study we performed an a priori power calculation for detecting a systemic measurement error of 2 pg/mL (at 80% power and an α-level of 0.05), we included groups consisting of (i) 32 patients for the main study of delayed processing of serum and plasma samples, and (ii) a validity cohort of 4 patients whose NfL concentrations in paired plasma and serum aliquots were determined repeatedly on four assay plates to determine inter-assay reliability^11^.

### Blood sampling

From all participants, peripheral venous blood was collected in Greiner Bio-One Vacuette serum and ethylenediaminetetraacetic acid (EDTA) tubes (GBO, Kremsmuenster, Austria). Blood vials were sent unaltered and at room temperature to the local biobank (Department of Laboratory Medicine, Medical University of Vienna) at different points in time depending on the group they were assigned to. Once in the biobank laboratory, samples were processed following standard operating procedures^12^. In brief, serum tubes were centrifuged after clotting had been completed at 1.884×g at room temperature. Serum was transferred to 500 µL polypropylene tubes and stored as 400 µL aliquots at − 70 °C until analysis. Plasma tubes were equally processed and stored without waiting for the material to coagulate before centrifugation.

### Sample handling for the delayed freezing-group

We drew a total of 4 serum and 4 plasma samples from each of the 32 participants in this group. Serum and plasma pairs from the same patient were stored at four different conditions before being sent to the local biobank for processing. The first serum and plasma tubes were sent to the local biobank immediately and at room temperature. The second serum and plasma tubes were placed in a refrigerator (temperature controlled at 4–8 °C) for 3 days before being sent to the local biobank. The third serum und plasma tubes were kept at room temperature for 3 days before being sent to the local biobank. The fourth serum and plasma tubes were kept at room temperature for 7 days before being sent to the local biobank.

### Sample handling for the control group measuring assay precision

In order to determine inter-assay reproducibility, we repeatedly measured NfL concentrations in 8 paired serum and plasma samples from 4 volunteers on four consecutive assay plates.

### Analysis of neurofilament light chain concentrations in serum and plasma

Serum and plasma aliquots were thawed for an hour at room temperature and analyzed by a blinded investigator. NfL concentrations in serum (sNfL) and plasma (pNfL) samples were determined using the Simoa Nf-light kits on a Simoa SR-X Analyzer following the manufacturer’s instructions (Quanterix, Billerica, MA, USA)^13^. In brief, thawed samples, calibrators and internal controls were equilibrated to room temperature, diluted (1:4) in sample diluent and transferred to the provided 96-well plates as duplicates. Well and plate assignment was designed so that all aliquots from the same participant were analyzed in the same run to avoid within-subject run-to-run variability. All assay plates (a total of 8) were run on a daily basis. Plates and samples were consecutively incubated with 20 µL of detector reagent, 25 µL of paramagnetic beads, and 100 μL streptavidin β-galactosidase. Over the course of these steps, plates were processed on the Simoa microplate incubator and Simoa microplate washer as per instructions. After final washing, pellets were allowed to dry for 10 min before transfer to the Quanterix SR-X analyzer for readout. As per usual convention, only samples yielding a coefficient of variance (CV) between paired replicates below 0.20 were included in this study.

### Statistical analyses

Statistical analysis was performed using SPSS 25.0 (SPSS Inc, Chicago, IL, USA). Categorical variables were expressed in frequencies and percentages, continuous variables were tested for Gaussian distribution by the Shapiro–Wilk test and displayed as mean and 95% CI or median and range as appropriate. Differences between sNfL and pNfL values after different freezing intervals were analyzed using repeated measurement ANOVA. A two-sided p-value of 0.05 was considered the level of significance. Reproducibility of sNfL measurement across different freezing intervals was assessed by calculating intraindividual standard deviations (iSD) and intraindividual covariances (iCOV). We also created Bland–Altman plots to visualize the agreement between different freezing intervals (Y-axis shows the difference between immediate freezing and different freezing intervals and the X-axis represents the average of these measures)^14^. Finally, intraclass correlation coefficients (ICC) were calculated. ICC calculation is based on a repeated measure ANOVA model using the variance among subjects, the variance among measurements, and the residual error variance^14^. An ICC of 1 represents perfect reproducibility, while an ICC above 0.9 is rated excellent for laboratory tests.

## Results

### Patient characteristics

Table 1 lists characteristics of participants in the main study setup (delayed freezing) and their mean NfL concentrations in serum (sNfL) and plasma (pNfL) at standard conditions (immediate processing). The mean age in this cohort was 40.2 years (95% CI 34.5–45.9), 38% of participants were female. The overall mean sNfL concentration was 14.5 pg/mL (95% CI 9.6–19.4), the mean pNfL concentration was 12.5 pg/mL (95% CI 8.6–16.5). Neurological diagnoses of patients included in this study were multiple sclerosis (n = 20), Parkinson’s disease (n = 6), Alzheimer disease (n = 4), and primary headache (n = 2).

**Table 1:** Patient characteristics.

Patient ID	Age	Sex	sNfL (pg/mL)	pNfL (pg/mL)
1	42	m	58.3	36.6
2	44	m	5.2	4.3
3	30	f	16.8	14.7
4	42	m	12.6	8.3
5	60	f	12.8	12.6
6	41	f	17.4	15.1
7	28	m	6.2	6.4
8	47	f	7.4	6.5
9	42	f	11.1	9.2
10	26	m	4.9	3.6
11	39	f	8.0	7.1
12	32	m	5.9	4.3
13	31	f	5.8	4.6
14	70	m	36.5	32.8
15	47	f	14.1	10.9
16	55	m	13.6	10.7
17	70	m	35.4	29.3
18	32	m	44.1	26.9
19	23	m	8.1	6.5
20	29	m	6.1	6.9
21	27	m	16.7	15.0
22	35	m	7.7	7.6
23	37	m	6.8	6.2
24	33	f	5.3	5.9
25	81	m	38.1	45.6
26	33	m	6.7	6.0
27	26	f	4.5	4.7
28	26	f	5.0	5.2
29	23	m	4.7	5.8
30	34	m	7.0	6.2
31	76	f	26.7	29.2
32	26	m	5.8	6.3

*f* female, *m* male, *pNfL* plasma neurofilament light chain, *sNfL* serum neurofilament light chain.

### NfL concentrations in serum and plasma withstand delayed processing up to 7 days

**Figure 1:**
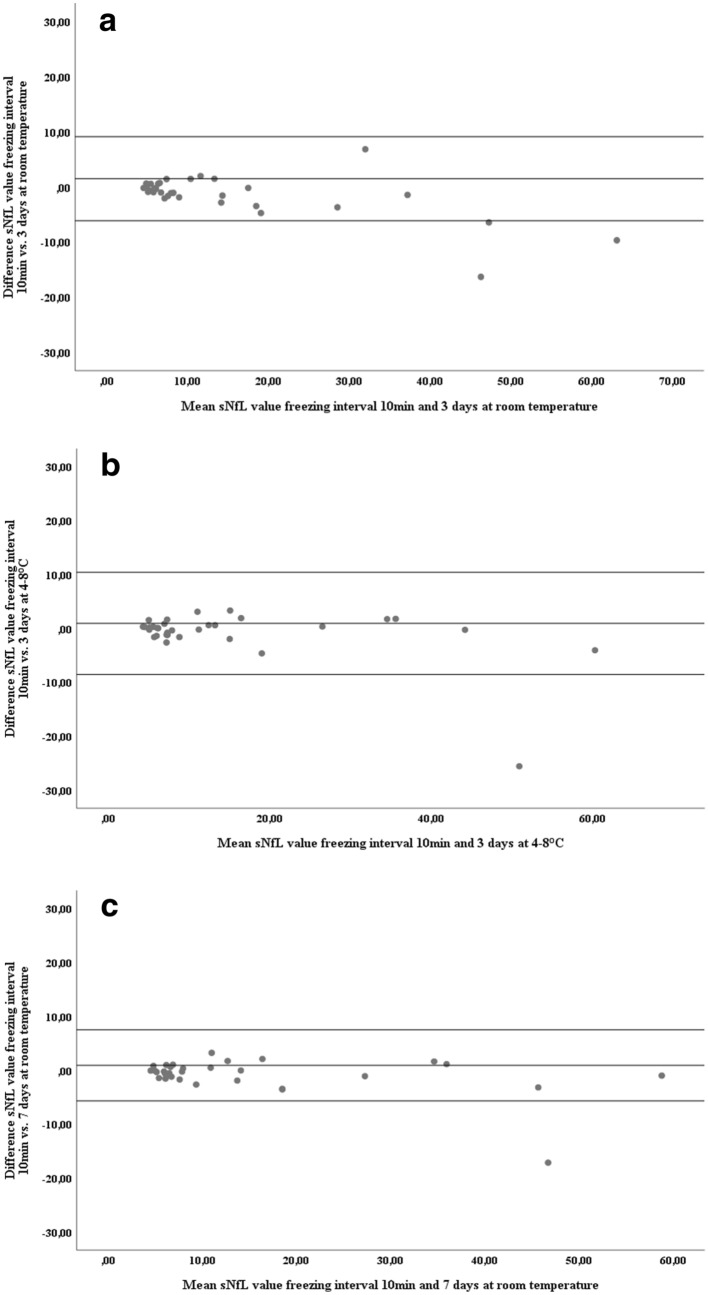
Differences in sNfL concentrations after different freezing intervals. Bland–Altman plot showing the differences in sNfL concentration between two groups of immediate processing and delayed freezing, (**a**) freezing after 10 min compared to 3 days at room temperature, (**b**) freezing after 10 min compared to 3 days at 4–8 °C, (**c**) freezing after 10 min compared to 7 days at room temperature. Single dots represent samples at two conditions with their mean concentrations on the x-axis and their difference in concentration on the y-axis. The three horizontal lines represent the mean difference (middle) and the mean difference plus 1.96 × SD of that difference (upper) and mean difference minus 1.96 × SD of that difference (lower).

**Figure 2:**
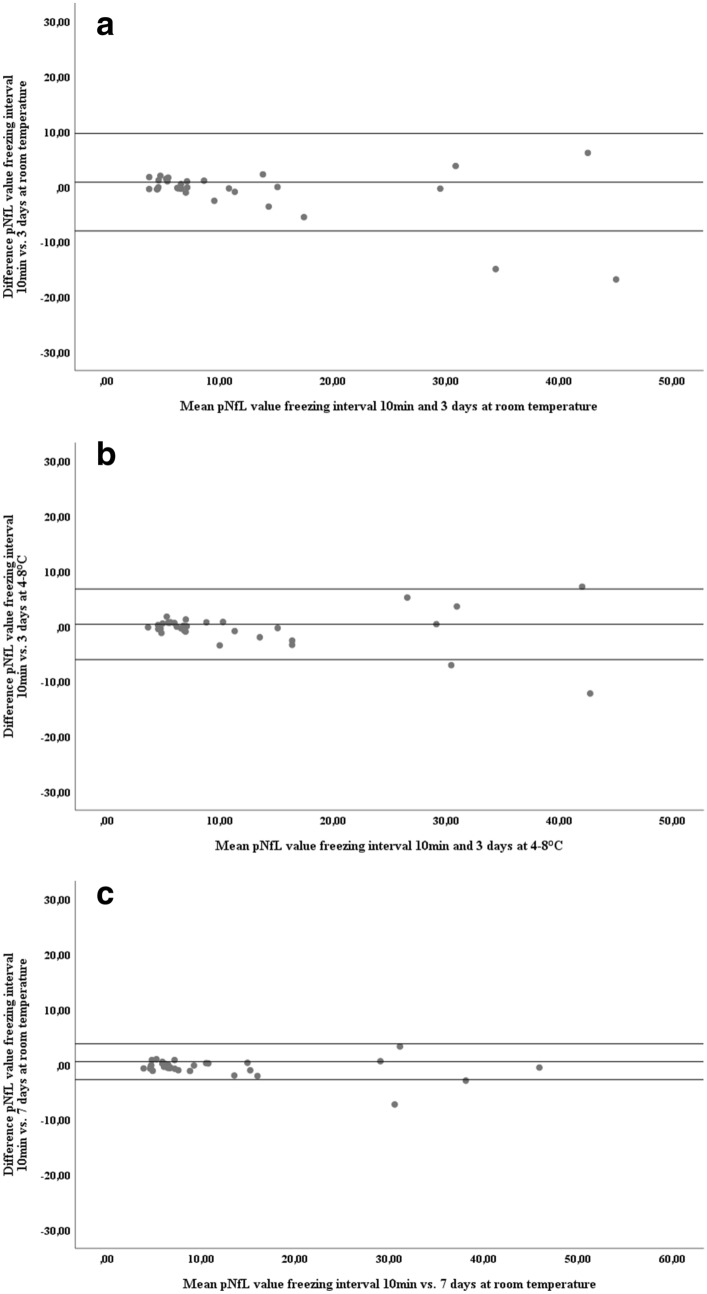
Differences in pNfL concentrations after different freezing intervals. Bland–Altman plot showing the differences in sNfL concentration between two groups of immediate processing and delayed freezing, (**a**) freezing after 10 min compared to 3 days at room temperature, (**b**) freezing after 10 min compared to 3 days at 4–8 °C, (**c**) freezing after 10 min compared to 7 days at room temperature. Single dots represent samples at two conditions with their mean concentrations on the x-axis and their difference in concentration on the y-axis. The three horizontal lines represent the mean difference (middle) and the mean difference plus 1.96xSD of that difference (upper) and mean difference minus 1.96xSD of that difference (lower).

Mean concentrations of sNfL and pNfL across all four pre-freezing intervals are shown in Table 2a (serum) and Table 2b (plasma). After storing of serum and blood tubes for 3 days at 4–8 °C, mean absolute differences compared to immediate freezing were 0.9 pg/mL for sNfL and 0.5 pg/mL for pNfL. Keeping samples stored for 3 days at room temperature yielded a mean absolute difference in concentration compared to immediate freezing of 1.4 pg/mL for sNfL and 0.8 pg/mL for pNfL. Ultimately, storing serum and blood vials for 7 days at room temperature resulted in a mean absolute difference in NfL concentrations compared to immediate freezing of 0.9 pg/mL for sNfL and 0.5 pg/mL for pNfL. Bland–Altman plots for these differences are shown in Fig. 1a–c (serum) and Fig. 2a–c (plasma). The reliability of sNfL and pNfL concentrations at the different pre-freezing intervals in comparison to immediate freezing are shown in Table 3a (serum) and Table 3b (plasma). For all three altered pre-freezing intervals, intraindividual variation (iCOV) was minimal (for samples stored for 7 days at room temperature; serum: 9.0%, plasma: 6.5%) and reproducibility close to perfect (for samples stored for 7 days at room temperature; serum: ICC = 0.99, plasma: ICC = 0.94). Separate analysis for the lowest and highest quartiles revealed still acceptable ICCs for sNfL and pNfL though intraclass correlation was better for serum.

**Table 2:** Comparison of NfL concentrations at different freezing intervals.

	[sNfL] freezing interval: immediate		[sNfL] freezing interval: 3 days RT		[sNfL] freezing interval: 3 days 4–8 °C		[sNfL] freezing interval: 7 days RT		p-value^a^
	Mean (pg/mL)	95% CI	Mean (pg/mL)	95% CI	Mean (pg/mL)	95% CI	Mean (pg/mL)	95% CI	
**(a)**									
Whole cohort	14.6	9.7–19.5	15.5	9.8–21.2	16.0	10.3–21.8	15.5	10.1–20.9	0.192
Lowest quartile	5.2	4.8–5.6	5.3	4.4–6.1	5.4	4.9–5.4	5.6	4.9–6.4	0.633
Highest quartile	34.2	22.6–45.7	36.8	20.8–52.7	38.5	23.7–53.3	36.8	23.5–50.1	0.422

	[pNfL] freezing interval: immediate		[pNfL] freezing interval: 3 days RT		[pNfL] freezing interval: 3 days 4–8 °C		[pNfL] freezing interval: 7 days RT		p-value^a^
	Mean (pg/mL)	95% CI	Mean (pg/mL)	95% CI	Mean (pg/mL)	95% CI	Mean (pg/mL)	95% CI	
**(b)**									
Whole cohort	12.0	8.1–15.9	12.5	8.3–16.8	12.8	8.1–17.5	12.5	8.4–16.6	0.385
Lowest quartile	4.8	4.1–5.5	4.8	4.4–5.3	4.3	3.7–4.8	5.0	4.4–5.5	0.071
Highest quartile	28.8	18.5–39.1	30.3	19.6–41.1	31.6	18.1–45.2	30.0	19.5–40.5	0.541

Mean values and 95% confidence intervals (95% CI) for (a) serum neurofilament light chain (sNfL) and (b) plasma neurofilament light chain (pNfL).

*CI* confidence interval, *pNfL* plasma neurofilament light chain concentration (pg/mL), *RT* room temperature, *sNfL* serum neurofilament light chain concentration (pg/mL).

^a^Calculated by repeated measurement ANOVA.

**Table 3:** Reliability of NfL concentrations at different freezing intervals compared to immediate freezing.

	Freezing interval: 3 days RT			Freezing interval: 3 days 4–8 °C			Freezing interval: 7 days RT		
	iSD [x10e3pg/L]	iCOV (%)	ICC	iSD [x10e3pg/L]	iCOV (%)	ICC	iSD [x10e3pg/L]	iCOV (%)	ICC
**(a) sNfL**									
Whole cohort	1.5	10.2	0.97	1.7	9.7	0.98	1.3	9.0	0.99
	0.3–2.6	8.2–12.2	0.94–0.99	0.9–2.6	6.5–12.8	0.96–0.99	0.5–2.0	6.5–11.5	0.97—0.99
Lowest quartile	0.5	7.0	0.90	0.4	9.5	0.91	0.5	8.1	0.90
	0.1–0.9	3.5–11.0	0.65–0.94	0.2–0.6	2.5–16.4	0.66–0.94	0.1–0.8	2.0–14.2	0.64–0.93
Highest quartile	3.4	11.1	0.92	4.3	10.2	0.94	2.7	8.5	0.95
	1.5–8.4	3.6–18.5	0.60–0.98	1.2–7.3	1.1–21.4	0.69–0.99	0.5–5.9	0.5–16.6	0.78–0.99
**(b) pNfL**									
Whole cohort	1.3	11.6	0.98	1.7	8.9	0.96	0.7	6.5	0.94
	0.6–2.0	8.1–15.1	0.96–0.99	0.6–2.7	6.5–11.2	0.92–0.98	0.4–1.1	4.8–8.2	0.90–0.99
Lowest quartile	0.4	16.3	0.90	0.6	7.8	0.90	0.5	7.6	0.88
	0.2–0.6	7.2–25.4	0.61–0.92	0.2–1.0	4.1–11.5	0.60–0.93	0.3–0.7	3.4–11.8	0.59–0.91
Highest quartile	3.4	14.2	0.91	4.5	11.9	0.86	1.7	5.6	0.84
	0.6–6.2	2.8–25.6	0.53–0.98	0.2–9.0	6.2–17.6	0.27–0.98	0.1–3.3	0.4–10.6	0.59–0.90

Mean values and 95% confidence intervals for (a) serum neurofilament light chain (sNfL) and (b) plasma neurofilament light chain (pNfL).

*ICC* intraclass correlation coefficient, *iCOV* intraindividual coefficient of variance, *iSD* intraindividual standard deviation, *pNfL* plasma neurofilament light chain, *RT* room temperature, *sNfL* serum neurofilament light chain.

### Inter-assay variability

Inter-assay variability within four serum and four plasma samples measured on four consecutive runs was perfect. For sNfL, the iSD was 0.6 pg/mL (range 0.5–1.2), the iCOV 4.8% (range 2.0–6.0) and the ICC 1.0 (range 0.99–1.00). For pNFL, the iSD was 1.6 pg/mL (range 0.8–2.7), the iCOV 12.7% (range 6.0–18.0) and the ICC 0.99 (range 0.90–1.00).

## Discussion

Neurofilament light chain is making a run at influencing how we monitor disease course and treatment in neurology. This study investigated a potential limitation for NfL research being constrained to immediate availability and accessibility of standardized sample processing, i.e., a biobanking procedure. We explored whether sNfL and pNfL concentrations remain stable when current standards in sample storage and handling cannot be met. Powered for detecting a measurement error of 2 pg/mL, we found that samples collected and stored at room temperature up to 7 days showed near-perfect stability of NfL concentrations in serum and a little less but still excellent stability in plasma (indicated by ICCs of 0.99 for serum and 0.94 for plasma). Our results are in line with two other studies reporting pre-analytical stability of *serum* NfL in smaller cohorts making this the largest study confirming this observation for *both* serum and plasma^10,11^. With this evidence at hand, we can recommend future practice in NfL research to allow delayed processing of serum or plasma samples. Native tubes can be stored or shipped for up to 7 days at room temperature and still yield reliable results for NfL concentrations.

Emphasis should be placed on the strengths and limitations of this study. First, we produced results for NfL measured in both serum and plasma allowing for a broad application of this knowledge. Second, particular focus was put on the analytical performance characteristics of the NfL assay itself, including precision and repeatability in serum and plasma samples. We provide data for inter-assay validity and found those results comparable to another large study investigating NfL and reporting assay precision^15^. Third, the results obtained from our study align well within the manufacturer statements regarding the limits of detection^13^. The mean lower limit of quantification is given as 0.316 pg/mL and the mean lower limit of detection as 0.0552 pg/mL. As for CVs reported by the manufacturer, the within run CVs ranged from 3.3–10.7 and the between run CVs from 5.3–11.7. Variation in our runs were comparably low as indicated by ICCs of 1.0 for serum and 0.99 for plasma. Fourth, the tested pre-freezing interval of 7 days at room temperature is of high practical relevance for the future. Once NfL enters clinical routine as a biomarker, we could expect peripheral hospitals to ship their native samples to a core facility.

There may be some limitations concerning the generalizability of this study. We used Greiner Bio-One Vacuette serum tubes as they are the most widely used product in our country. Other collection systems may produce different results. That being said, this issue per se applies to every biomarker study. Furthermore, the validity of our results depends on meticulous quality control in a specialized biomarker laboratory. A real-world setting in a busy lab is expectedly more prone to accidental protocol deviation, human error, and other preanalytical errors such as hemolysis or the fact that various analyses are performed on a Quanterix reader for different analytes at the same time. Moreover, our results relate to a population showing sNfL concentrations ranging from 4.5 to 58.3 pg/mL and may not be applied to higher concentrations.

A few remarks should be made on comparing the analytical performance of serum and plasma samples in this pre-analytical study. First, the mean concentration of NfL in plasma was 10% lower (95% CI 5–16) than in serum which compares well to another investigation^10^. Second, our results suggest that reliability for prolonged storage was slightly better in serum than plasma (ICC 0.99 vs. 0.94). Possible explanations for this observation may constitute an interference of persisting coagulation factors through EDTA. Therefore, one may conclude that native serum samples may be slightly more suitable for shipment or any case of delayed processing for up to 7 days.

In summary, we demonstrated how sNfL and pNfL concentrations remain stable if sample are left at room temperature up to 7 days before being processed at standardized laboratory conditions. Our results may help NfL to further evolve as a biomarker in neurological disease as future protocols can account for pre-analytical stability in terms of delayed sample storage.

## Abbreviations

sNfL: Serum neurofilament light chain
pNfL: Plasma neurofilament light chain
NfL: Neurofilament light chain
EDTA: Ethylenediaminetetraacetic acid
CI: Confidence interval
iSD: Intraindividual standard deviation
iCOV: Intraindividual coefficient of variation
ICC: Intraclass correlation coefficient
