# Author Correction: Seven day pre-analytical stability of serum and plasma neurofilament light chain

**DOI:** 10.1038/s41598-022-10916-3

**Published:** 2022-04-20

**Authors:** Patrick Altmann, Markus Ponleitner, Paulus Stefan Rommer, Helmuth Haslacher, Patrick Mucher, Fritz Leutmezer, Axel Petzold, Christoph Wotawa, Rupert Lanzenberger, Thomas Berger, Henrik Zetterberg, Gabriel Bsteh

**Affiliations:** 1 Department of Neurology, Medical University of Vienna, Waehringer Guertel 18-20, 1090 Vienna, Austria; 2 Department of Laboratory Medicine, Medical University of Vienna, Vienna, Austria; 3 Department of Neuroinflammation, UCL Queen Square Institute of Neurology, London, UK; 4 Department of Psychiatry and Psychotherapy, Medical University of Vienna, Vienna, Austria; 5 Department of Neurodegenerative Disease, UCL Queen Square Institute of Neurology, London, UK; 6 UK Dementia Research Institute at UCL, London, UK; 7 Department of Psychiatry and Neurochemistry, Institute of Neuroscience and Physiology, The Sahlgrenska Academy at the University of Gothenburg, Mölndal, Sweden; 8 Clinical Neurochemistry Laboratory, Sahlgrenska University Hospital, Mölndal, Sweden

Correction to: *Scientific Reports* 10.1038/s41598-021-90639-z, published online 26 May 2021

The original version of this Article contained errors, where the intraindividual coefficient of variation (iCOV) was calculated incorrectly.

As a result, in the Results section under the subheading ‘NfL concentrations in serum and plasma withstand delayed processing up to 7 days’,

“For all three altered pre-freezing intervals, intraindividual variation (iCOV) was minimal (for samples stored for 7 days at room temperature; serum: 1.2%, plasma: 1.3%) and reproducibility close to perfect (for samples stored for 7 days at room temperature; serum: ICC = 0.99, plasma: ICC = 0.94).”

now reads:

“For all three altered pre-freezing intervals, intraindividual variation (iCOV) was minimal (for samples stored for 7 days at room temperature; serum: 9.0%, plasma: 6.5%) and reproducibility close to perfect (for samples stored for 7 days at room temperature; serum: ICC = 0.99, plasma: ICC = 0.94).”

Additionally, under the subheading ‘Inter‑assay variability’ of the Results section,

“For sNfL, the iSD was 0.6 pg/mL (range 0.5–1.2), the iCOV 0.1% (range 0.0–0.1) and the ICC 1.0 (range 0.99–1.00). For pNFL, the iSD was 1.6 pg/mL (range 0.8–2.7), the iCOV 0.1% (range 0.1–0.2) and the ICC 0.99 (range 0.90–1.00).”

now reads:

“For sNfL, the iSD was 0.6 pg/mL (range 0.5–1.2), the iCOV 4.8% (range 2.0–6.0) and the ICC 1.0 (range 0.99–1.00). For pNFL, the iSD was 1.6 pg/mL (range 0.8–2.7), the iCOV 12.7% (range 6.0–18.0) and the ICC 0.99 (range 0.90–1.00).”

Furthermore, Table 3 contained errors in the “iCOV (%)” columns for “Freezing intervals: 3 days RT”, “Freezing intervals: 3 days 4-8 °C” and “Freezing intervals: 7 days RT”.

The original Table 3 and accompanying legend appear below.

**Table 3:** Reliability of NfL concentrations at different freezing intervals compared to immediate freezing.

	Freezing interval: 3 days RT			Freezing interval: 3 days 4-8 °C			Freezing interval: 7 days RT		
	iSD [pg/mL]	iCOV (%)	ICC	iSD [pg/mL]	iCOV (%)	ICC	iSD [pg/mL]	iCOV (%)	ICC
**(a) sNfL**									
Whole cohort (and range)	1.5	1.2	0.97	1.7	1.2	0.98	1.3	1.2	0.99
	0.3–2.6	1.1–1.3	0.94–0.99	0.9–2.6	1.1–1.2	0.96–0.99	0.5–2.0	1.1–1.3	0.97–0.99
Lowest quartile (and range)	0.5	1.2	0.90	0.4	1.2	0.91	0.5	1.2	0.90
	0.1–0.9	1.0–1.3	0.65–0.94	0.2–0.6	1.2–1.3	0.66–0.94	0.1–0.8	1.1–1.4	0.64–0.93
Highest quartile (and range)	3.4	1.2	0.92	4.3	1.2	0.94	2.7	1.2	0.95
	1.5–8.4	1.0–1.4	0.60–0.98	1.2–7.3	1.0–1.3	0.69–0.99	0.5–5.9	1.1–1.4	0.78–0.99
**(b) pNfL**									
Whole cohort (and range)	1.3	1.2	0.98	1.7	1.1	0.96	0.7	1.3	0.94
	0.6–2.0	1.1–1.3	0.96–0.99	0.6–2.7	1.1–1.2	0.92–0.98	0.4–1.1	1.2–1.3	0.90–0.99
Lowest quartile (and range)	0.4	1.2	0.90	0.6	1.1	0.90	0.5	1.2	0.88
	0.2–0.6	1.1–1.3	0.61–0.92	0.2–1.0	0.9–1.3	0.60–0.93	0.3–0.7	1.1–1.3	0.59–0.91
Highest quartile (and range)	3.4	1.2	0.91	4.5	1.1	0.86	1.7	1.3	0.84
	0.6–6.2	1.0–1.3	0.53–0.98	0.2–9.0	0.9–1.4	0.27–0.98	0.1–3.3	1.1–1.4	0.59–0.90

Mean values and 95% confidence intervals for (a) serum neurofilament light chain (sNfL) and (b) plasma neurofilament light chain (pNfL).

*ICC* intraclass correlation coefficient, *iCOV* intraindividual coefficient of variance, *iSD* intraindividual standard deviation, *pNfL* plasma neurofilament light chain, *RT* room temperature, *sNfL* serum neurofilament light chain.

The original Article has been corrected.

